# Associations between elevated kidney and liver biomarker ratios, metabolic syndrome and all-cause and coronary heart disease (CHD) mortality: analysis of the U.S. National Health and Nutrition Examination Survey (NHANES)

**DOI:** 10.1186/s12872-021-02160-w

**Published:** 2021-07-26

**Authors:** Akinkunle Oye-Somefun, Jennifer L. Kuk, Chris I. Ardern

**Affiliations:** School of Kinesiology and Health Science, 222A Bethune College, York University, 4700 Keele Street, Toronto, ON M3J1P3 Canada

**Keywords:** Biomarkers, Kidney function, Metabolic syndrome, Mortality, NHANES, Obesity

## Abstract

**Background:**

We examined the relationship between ratios of select biomarkers of kidney and liver function on all-cause and coronary heart disease (CHD) mortality, both in isolation, and in combination with metabolic syndrome (MetS), among adults (20 + years, n = 10,604).

**Methods:**

Data was derived from the U.S. National Health and Nutrition Examination Survey (1999–2016) including public-use linked mortality follow-up files through December 31, 2015.

**Results:**

Select biomarker ratios of kidney (UACR or albuminuria and BUN-CR) and liver (AST-ALT and GGT-ALP) function in isolation and in combination with MetS were associated with all-cause and CHD mortality. Compared to individuals with neither elevated biomarker ratios nor MetS (HR = 1.00, referent), increased risk of all-cause mortality was observed in the following groups: MetS with elevated UACR (HR, 95% CI = 2.57, 1.99–3.33), MetS with elevated AST-ALT (HR = 2.22, 1.61–3.07), elevated UACR without MetS (HR = 2.12, 1.65–2.72), and elevated AST-ALT without MetS (HR = 1.71, 1.35–2.18); no other biomarker ratios were associated with all-cause mortality. For cause-specific deaths, elevated risk of CHD mortality was associated with MetS with elevated UACR (HR = 1.67, 1.05–2.67), MetS with elevated AST-ALT (HR = 2.80, 1.62–4.86), and elevated BUN-CR without MetS (HR = 2.12, 1.12–4.04); no other biomarker ratios were associated with CHD mortality.

**Conclusion:**

Future longitudinal studies are necessary to examine the utility of these biomarker ratios in risk stratification for chronic disease management.

**Supplementary Information:**

The online version contains supplementary material available at 10.1186/s12872-021-02160-w.

## Introduction

Current estimates are that one-third of all adults in the United States (U.S.) have the metabolic syndrome (MetS)—a cluster of cardiometabolic conditions that are leading contributors to an elevated risk for cardiovascular disease (CVD) and type II diabetes [1–3]. While the major complications of MetS are now well defined, a number of related consequences have been identified, including an increased risk of fatty liver disease [4–6], renal failure [7–9], and low grade chronic inflammation [10–15], that collectively increase risk of premature death [16–20].

Over the past three decades, the contribution of a host of biomarkers to morbidity and mortality has received increasing attention [5, 6, 8–10, 16–25]. Included in these are validated molecular biomarkers, such as liver enzymes alanine aminotransferase (ALT), aspartate aminotransferase (AST), alkaline phosphatase (ALP), and gamma-glutamyl transferase (GGT), that serve as reliable indicators of past exposures and future adverse cardiometabolic abnormalities [22–25]. Liver enzymes are routinely evaluated—in isolation, or less frequently as ratios—when investigating suspected biliary conditions, or liver conditions such as non-alcoholic fatty liver disease (NAFLD), and alcoholic liver disease (ALD) [5, 6, 22–25]. Similarly, surrogates of kidney disease such as urinary albumin, urinary creatinine, blood urea nitrogen, and serum creatinine, help to underscore the interrelated nature of renal pathology [16–21].

Until recently, little was known about the co-occurrence of these biomarkers in cardiometabolic disease, but in recent years, the application of validated molecular biomarkers in the study of cardiovascular and metabolic pathways has been increasing [17, 19–25]. The ratios of these biomarkers have therefore been proposed as a way to advance the risk assessment process [5, 15]. Of note, albuminuria—which can be characterized as elevated urinary albumin to creatinine (UACR) ratio—is recognized by the World Health Organization as a stand-alone component within its criteria for MetS [1, 4]. Elevated kidney biomarkers suggest vascular injury of the kidneys and systemic endothelial dysfunction [8, 9, 16–21], whereas elevated liver enzymes may reflect liver dysfunction related to alcohol abuse [22], insulin resistance [23], or impaired perfusion [10, 11, 24], which has also been linked to venous congestion and renal dysfunction [9, 20]. Given the interrelationship, endothelial dysfunction [9], impaired perfusion or cardiac output [10], and systemic inflammation [11] are a few of the common underlying mechanisms of abnormal kidney or liver biomarkers [10, 16, 17].

To date, the use of various kidney or liver biomarker ratios to predict mortality, are nevertheless lacking in large-scale or population-based studies involving cardiovascular disease endpoints. Moreover, the combined effect of biomarkers and MetS on mortality has not been fully evaluated, however, even as emerging evidence now supports their use in diagnostic algorithms to help in the identification of high-risk groups [5, 20, 24, 25, 29]. Building on previous studies on kidney [17–21, 25] and liver [15, 16, 22–25] biomarkers to explore the utility of co-occurring biomarkers ratios may provide new insight into their independent and joint effects on mortality. Previous analysis of the U.S. National Health and Nutrition Examination Survey (NHANES) have also relied on the Beckman CX3 using a Jaffé reaction method of assessment for serum and urine creatinine [26, 27], which has recently been updated with a correction factor, and warrants re-analysis [27].

The purpose of this study is to examine the independent and joint associations of kidney and liver biomarker ratios with MetS on all-cause and CHD mortality, using public-use linked mortality files for the U.S. NHANES. We hypothesize that elevated ratios of biomarkers both in isolation, and in combination with MetS will be associated with all-cause and cause-specific deaths, compared to the referent groups—adults with (1) no MetS nor elevated ratios, and; (2) prevalent MetS only.

## Methods

### Database

Data for this study was derived from multiple cycles of the cross-sectional Continuous NHANES. The Continuous NHANES uses a complex, multistage, probability cluster design in order to yield a nationally representative survey of the health and nutritional status of the non-institutionalized civilian population in the U.S., full details on which are described in the NHANES survey methods and analytic guidelines [30]. Ethics approval was obtained from the National Center for Health Statistics Research Ethics Review Board (ERB) for NHANES 1999–2004 (Protocol #98-12), NHANES 2005–2010 (Protocol #2005-06), NHANES 2011–2016 (Protocol #2011-17) on which data for this analysis was used. Informed, written consent was obtained from all participants. This study is an analysis of NHANES publicly available anonymized data, and thus, does not require further ethical review from the York University institutional review board.

### Study sample

**Fig. 1:**
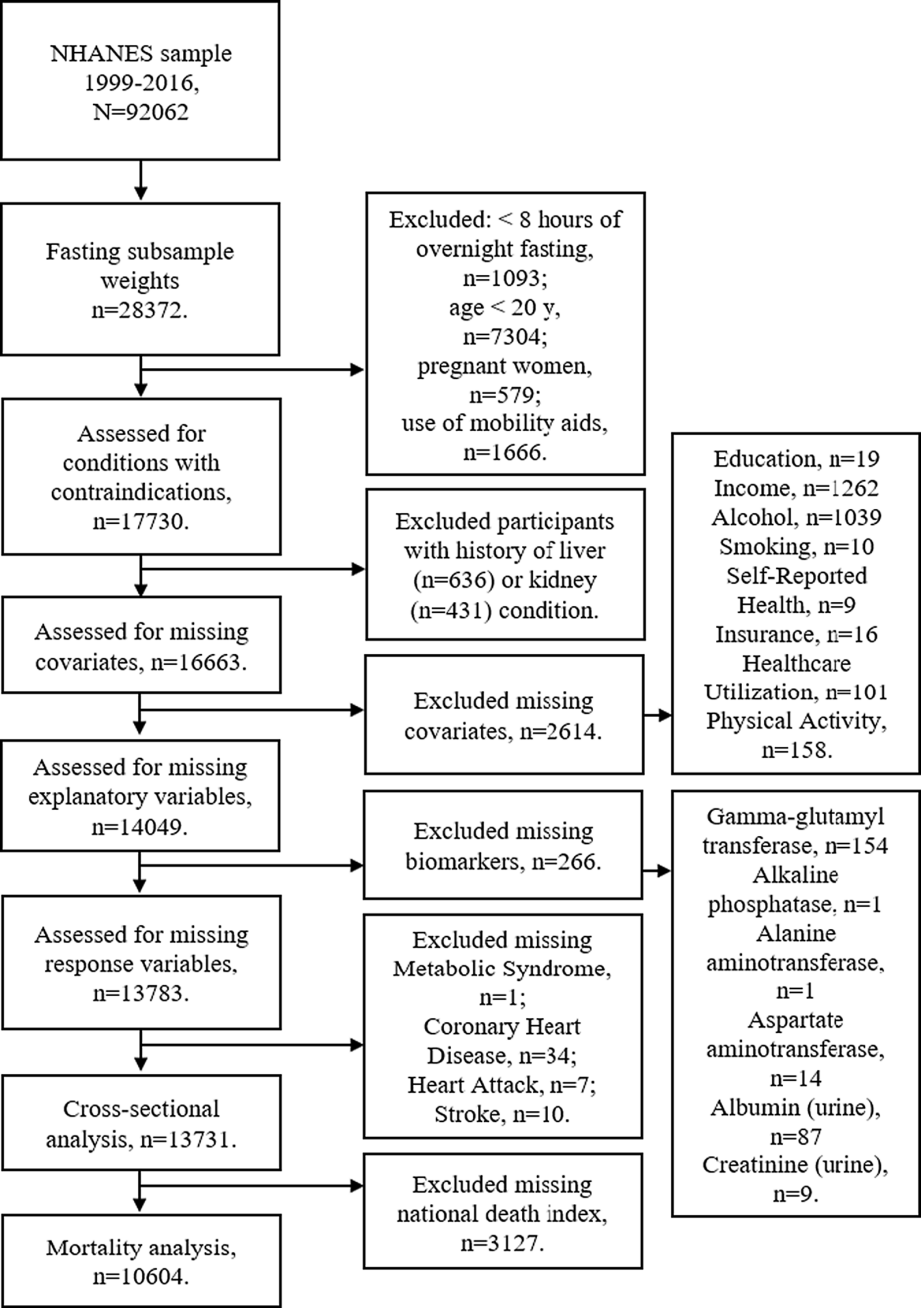
Flowchart showing the data collection for the analytic sample

The original sample is an amalgamation of nine consecutive cycles collected in 2-year increments from 1999 through 2016 of the continuous NHANES. The initial unweighted sample size was 92,062 (Fig. 1). A final analytical sample of 13,731 was obtained for the cross-sectional analysis after listwise exclusion of individuals under 20 years of age (n = 42,550), women who were pregnant (n = 1486), those reporting (yes/no) use of mobility aids (n = 4581), cases with less than eight hours of overnight fasting (n = 21,745), cases without fasting sampling weights (n = 3970), those reporting (yes/no) any liver (n = 636) or kidney (n = 431) conditions, and missing data for covariates.

### Data collection

Information on demographic, lifestyle, physical measurements, and standard biochemistry profiles were collected. Standardized physical examinations, and laboratory tests, were performed in controlled environments [27]. Trained field health technicians conducted interviews at each mobile exam center (MEC), performed physical examinations (e.g., height, weight, waist circumference and blood pressure), as well as biospecimens (blood and urine) collection, which includes screening for pregnancy, storing, and shipping of biospecimens [27].

### Standard biochemistry profile

The NHANES Laboratory Method Files offer detailed description of the biological specimen collection, storage, and laboratory methods used [27]. Clinical biospecimens were often analyzed using either a Beckman Synchron LX20 prior to the 2007–2008 cycle, or a Beckman UniCel DxC 800 Synchron after the 2007–2008 cycle because of an instrumentation change in 2007 [27]. Coefficient of variation (CV) for all laboratory biomarkers were assessed using univariate analysis, and deemed to be within acceptable range (< 10%) [27, 31, 32].

Serum ALT and ALP activity were determined using a kinetic rate method, while an enzymatic rate method was used to determine AST and GGT activity [27]. An enzymatic conductivity rate method was also used to determine the concentration of blood urea nitrogen (BUN) [27]. Serum creatinine (CR) concentration was determined using a Jaffé rate method (kinetic alkaline picrate); however, CR from the periods 1999–2000 and 2005–2006 were determined to have bias due to a lack of standardized methods, or calibration [26]. In order to adjust CR for those cycles, a correction factor was recommended [27].

Glucose concentration was determined using a hexokinase-mediated reaction method, while a timed-endpoint method was used to determine the concentration of serum triglycerides [27]. Despite changes in the lab site, method, and instrumentation to measure triglycerides across cycles, no adjustments were deemed necessary as a result of these changes.

High density lipoprotein (HDL) cholesterol was analyzed in 1999–2002 primarily by heparin manganese precipitation or by direct HDL immunoassay depending on the participant age and amount of specimen [27]. Beginning in 2003, all specimens were analyzed using the direct HDL cholesterol immunoassay method, and in 2007–2008, a new laboratory performed the direct HDL cholesterol method on a different instrument [27]. HDL cholesterol values for 1999–2000, 2001–2002 and 2005–2006 were therefore adjusted to account for changes in instrumentation and methods using the CDC correction factor set out by Solomon Park Research Laboratories (Kirkland, WA) quality controls correction factor [27]. With the exception of HDL values for 1999–2002, which could not be evaluated, all laboratories were within the 5% maximum allowable bias for HDL [27].

Urinary albumin (UA) was measured using a solid-phase fluorescent immunoassay, which is a non-competitive, double-antibody method [27]. A Beckman Synchron CX3 Clinical Analyzer Jaffé rate reaction method was used to measure urinary creatinine (UCR) by enzymatic method prior to 2007 [27]. Due to an instrument change in 2007–2008, UCR was measured using Roche/Hitachi Modular P Chemistry Analyzer [27]. In accordance with recommendations, a piecewise regression approach was used to adjust UCR prior to 2007 to be comparable with UCR from 2007 onwards [27].

### Public-use linked mortality files (LMF)

The outcomes for this study were all-cause and CHD mortality. The public-use versions of the LMF include a limited set of mortality variables for adult participants only after files were processed to reduce the participant disclosure risk. The public-use LMF provides mortality follow-up data from the date of survey participation through the end of the mortality period—December 31, 2015 (sample size for this analysis, n = 10,604). Data collected from the National Center for Health Statistics (NCHS) has linked several population surveys with death certificate records from the National Death Index (NDI) [35]. All-cause mortality included all known as well as unknown causes, while CHD mortality was defined as diseases of the heart using international classification of diseases coding (ICD-9 and ICD-10), including: diseases of heart (I00–I09, I11, I13, I20–I51), acute rheumatic fever and chronic rheumatic heart diseases (I00–I09), hypertensive heart disease (I11), hypertensive heart and renal disease (I13), ischemic heart diseases (I20–I25), acute myocardial infarction (I21–I22), other acute ischemic heart diseases (I24), other forms of chronic ischemic heart disease (I20, I25), and atherosclerotic cardiovascular disease (I25.0), all other forms of chronic ischemic heart disease (I20, I25.1–I25.9), and other heart diseases (I26–I51) [35].

### Markers of cardiometabolic risk

Screening for MetS included the presence of three or more of the following five risk factors: high triglycerides (≥ 150 mg/dl); reduced high-density lipoprotein cholesterol (≤ 40 mg/dl [males] and ≤ 50 mg/dl [females]); high fasting glucose (≥ 100 mg/dl or diabetes medications); high blood pressure (systolic ≥ 130 mmHg or diastolic ≥ 85 mmHg, or hypertensive medications), and; high waist circumference (≥ 102 cm [males] and ≥ 88 cm [females]) [1, 4]. Comorbid conditions (CVD, diabetes, and hypertension) were determined using self-reports of medical conditions informed by a doctor or health professional [27].

For serum biomarkers, the *Johns Hopkins Hospital’s Harriet Lane Handbook* was consulted for the general population or sex-specific reference ranges [33]. For urinary biomarkers, the CDC reference ranges were consulted [27]. Biomarkers were selected and computed as ratios on the basis of previous research that established a link with insulin resistance or MetS [1, 4, 6, 15, 23]. Because biomarkers may vary by clinical laboratory settings, instrumentation, or method, a 90th percentile cut-off level was deemed as an acceptable “high-normal” to have consistency between biomarkers and sufficient group sizes for the pooled data [34]; survey cycles were pooled for the purpose of the current analysis. Elevated biomarker ratios were therefore categorized using the 90th percentile cut-offs for the pooled data as follows: BUN-CR (≥ 21.21 mg/dL), GGT-ALP (≥ 0.71 U/L), and AST-ALT (≥ 1.47 U/L). A urinary albumin to creatinine ratio (UACR) was determined by NHANES laboratory protocol [27]. Established threshold for low UACR (normoalbuminuria (< 30 mg/g)) and elevated UACR (albuminuria (≥ 30 mg/g)), were used [1, 4].

Participants were subsequently cross-classified into four discrete groups: (1) non-MetS with non-elevated biomarker ratio; (2) non-MetS with elevated biomarker ratio; (3) MetS with non-elevated biomarker ratio, and; (4) MetS with elevated biomarker ratio.

### Covariates

All analyses were adjusted for age, sex, ethnicity (Mexican American, other Hispanic, non-Hispanic White, non-Hispanic Black, Other/Multi-Ethnic), education, income status (a ratio of household income to the related poverty cut-off level), alcohol status (“*had at least 12 drinks in a year*”), lifetime smoking status (“*had at least 100 cigarettes in life*”), self-reported general health, health insurance coverage, healthcare utilization (frequency of hospital visits), physical activity (PA), and survey collection year [27]. Weekly minutes of moderate or vigorous-intensity PA was ascertained and used to categorize participants as “low” (< 150 min/week), or “high” (≥ 150 min/week), consistent with current PA guidelines [27].

### Statistical analysis

Our first objective was to determine the prevalence and cross-sectional association between biomarkers ratios and MetS with prevalent CVD. In an initial step, socio-demographic, health, and behavioral characteristics of the sample were compared between those *with* versus *without* MetS using t-tests and chi-squared analysis, as appropriate. Biomarker ratios (mean ± SEM), and the prevalence of elevated biomarker ratios, were compared between groups with and without MetS using t-tests and chi-square, respectively. Unadjusted and fully adjusted (for covariates: age, sex, ethnicity, education, income, alcohol, smoking status, self-reported general health, health insurance coverage, frequency of hospital visits, physical activity, and survey collection year) logistic regression models were used to assess the independent and joint associations of the MetS/biomarker groups with CVD in a sensitivity analysis (n = 13,731). Using these groups, the proportional hazards assumption was subsequently assessed and upheld. For the mortality analysis (n = 10,604), a final series of Cox proportional hazards regression models (semi-parametric) were developed to examine the unadjusted and fully adjusted (for covariates) relationship for each discrete category of biomarkers with the referent (i.e., no MetS nor elevated ratios, and; prevalent MetS) and all-cause or CHD mortality (HR = 1.00, referent). A hazard ratio greater than the null value (HR = 1.00) suggests a higher risk for the event (or lower risk when less than the null). Data analysis was performed with SAS software version 9.4 (Cary, NC, U.S.A.), weighted with the master survey weights to ensure national representativeness of the data. Statistical significance was set at alpha = 0.05.

## Results

Table 1 shows the sociodemographic and clinical characteristics among participants with and without MetS (*p* < 0.01). Of note, MetS was more prevalent in older age, and had a higher proportion of males, Mexican Americans, and non-Hispanic Whites, compared to the non-MetS group. The MetS group had a higher proportion of individuals without college education and lower incomes, compared to the non-MetS group. The MetS group also had a higher proportion of smokers, more frequent hospital visits, insurance coverage, lower self-rated health, and more inactivity, compared to the non-MetS group.

**Table 1:** Characteristics of Adults (20 + years), NHANES 1999–2016

Analytic sample	Total	MetS	
(n = 13,731)		No	Yes
MetS (%)		69.11 (0.53)	30.89 (0.53)
Age (y): Mean (SEM)	45.76 (0.25)	43.23 (0.31)	51.42 (0.30)
Sex: Males (%)	49.76 (0.39)	48.75 (0.52)	52.0 (0.90)
Ethnicity			
Mexican American (%)	7.66 (0.54)	7.26 (0.50)	8.53 (0.75)
Other Hispanic (%)	5.00 (0.54)	5.02 (0.51)	4.96 (0.71)
Non-Hispanic White (%)	71.31 (1.11)	70.32 (1.11)	73.53 (1.36)
Non-Hispanic Black (%)	10.20 (0.60)	10.99 (0.65)	8.42 (0.59)
Other Ethnicity (%)	5.83 (0.31)	6.40 (0.37)	4.56 (0.42)
Education			
Less than High School (%)	15.80 (0.54)	14.26 (0.61)	19.25 (0.76)
High School Graduate (%)	23.21 (0.64)	21.19 (0.74)	27.71 (1.03)
College Education (%)	60.99 (0.93)	64.54 (1.04)	53.04 (1.17)
Income ratio: mean (SEM)	3.09 (0.03)	3.13 (0.04)	3.01 (0.04)
< 1.3 (%)	18.94 (0.67)	18.30 (0.76)	20.39 (0.87)
1.3–3.5 (%)	36.33 (0.70)	35.67 (0.78)	37.82 (1.06)
> 3.5 (%)	44.73 (0.95)	46.04 (1.04)	41.80 (1.18)
Acohol: Yes (%)	76.85 (0.78)	78.95 (0.76)	72.15 (1.11)
Smoking: Yes (%)	46.09 (0.75)	44.46 (0.87)	49.73 (1.05)
Hospital visits			
None (%)	17.52 (0.46)	18.58 (0.60)	15.14 (0.68)
1 visit (%)	20.12 (0.44)	21.39 (0.49)	17.27 (0.81)
2–3 visits (%)	29.97 (0.47)	30.28 (0.54)	29.28 (0.95)
4–9 visits (%)	22.53 (0.42)	20.69 (0.48)	26.64 (0.86)
≥ 10 visits (%)	9.87 (0.33)	9.06 (0.35)	11.67 (0.70)
Insurance: Yes (%)	81.95 (0.61)	81.12 (0.72)	83.80 (0.73)
Health status: poor/fair (%)	13.08 (0.40)	10.28 (0.41)	19.32 (0.69)
Physical activity minutes per week: mean (SEM)	609.51 (15.06)	645.77 (17.06)	528.38 (20.28)
< 150 min/week MVPA (%)	40.47 (0.66)	37.68 (0.69)	46.73 (1.12)
Cardiovascular disease (%)	5.14 (0.25)	3.76 (0.24)	8.25 (0.49)
Coronary heart disease (%)	2.55 (0.16)	1.97 (0.18)	3.86 (0.32)
Heart attack (%)	2.39 (0.16)	1.69 (0.16)	3.95 (0.33)
Stroke (%)	1.86 (0.15)	1.33 (0.14)	3.03 (0.32)
UACR ratio: mean (SEM)	19.35 (1.31)	13.49 (0.71)	32.57 (4.06)
Elevated (%)	7.76 (0.27)	5.61 (0.29)	12.56 (0.61)
BUN-CR ratio: mean (SEM)	15.07 (0.08)	14.87 (0.09)	15.50 (0.12)
Elevated (%)	11.39 (0.39)	10.48 (0.42)	13.44 (0.69)
GGT-ALP ratio: mean (SEM)	0.41 (0.01)	0.38 (0.01)	0.49 (0.01)
Elevated (%)	10.00 (0.33)	7.88 (0.34)	14.75 (0.75)
AST-ALT ratio: mean (SEM)	1.08 (0.01)	1.12 (0.01)	0.98 (0.01)
Elevated (%)	10.10 (0.34)	12.26 (0.44)	5.27 (0.36)

Prevalence of elevated biomarker ratios, including socio-demographic, health, and behavioral characteristics compared between adults with versus without MetS using t-tests and chi-squared analysis, as appropriate. All *P* < .01; chi-square for frequencies, and independent sample t-test for continuous measures. Values in parentheses represent SEM for continuous measures or SE for proportions

Overall, the prevalence of elevated biomarker ratios for MetS and non-MetS groups showed that the MetS group had a higher proportion with elevated kidney biomarkers (UACR or albuminuria, and BUN-CR), and elevated liver biomarkers GGT-ALP. As well, the MetS group tended to have higher mean ratios for UACR, BUN-CR, GGT-ALP, compared to the non-MetS group.

### All-cause mortality

**Fig. 2:**
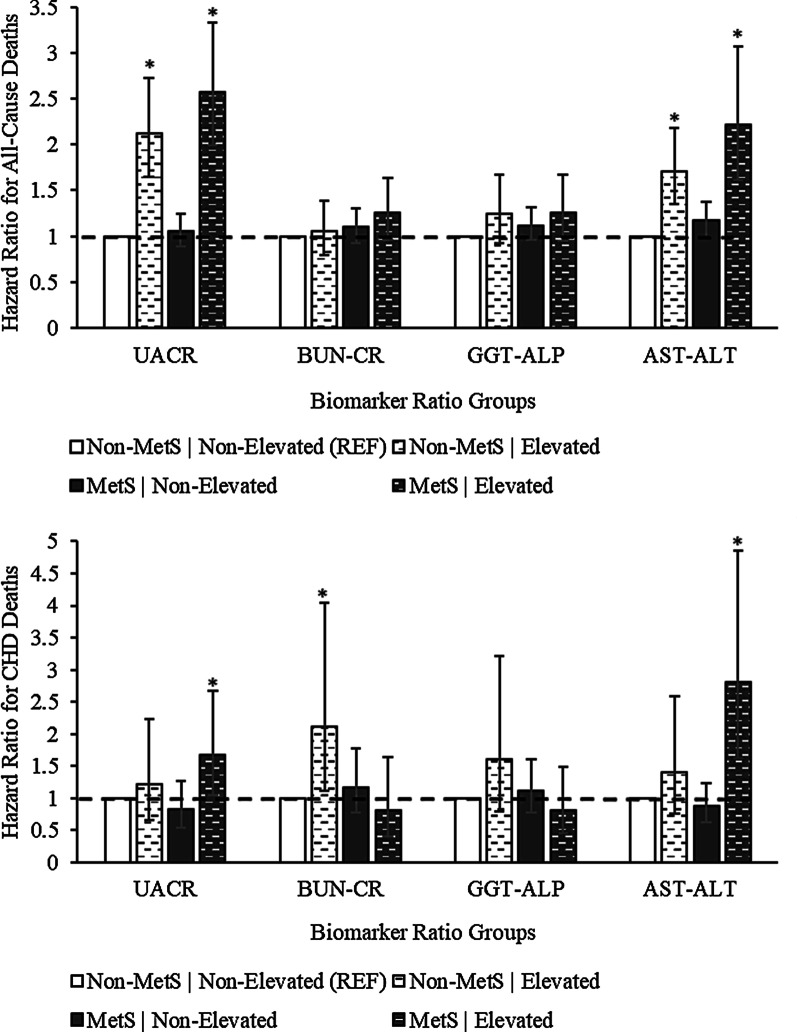
Relationships between groups of biomarker ratios and deaths due to all-causes and coronary heart disease. Data derived from NHANES 1999–2015; referent group has neither elevated biomarker ratio nor MetS. Adjusted for age, sex, ethnicity, education, income ratio, alcohol use, smoking, hospital visits, insurance coverage, self-rated health, and physical activity. Statistical significance was denoted with an asterisk (*) to represent a statistically significant association with the event—compared to the referent group

Results of the unadjusted and fully adjusted (for covariates) Cox proportional hazards models of the association between all-cause mortality and co-occurrence of MetS with kidney, or liver, biomarker ratios are presented in tables (see Additional file 1). In the fully adjusted model of the analytic sample (Fig. 2), elevated biomarker ratio with prevalent MetS (elevated UACR: HR, 95% CI = 2.57, 1.99–3.33, and elevated AST-ALT: HR = 2.22, 1.61–3.07) was positively associated with all-cause mortality, compared to the referent group—that is, the group with neither elevated biomarker ratio nor MetS. Positive associations with all-cause mortality were also observed for these elevated biomarker ratios without MetS (elevated UACR: HR = 2.12, 1.65–2.72, and elevated AST-ALT: HR = 1.71, 1.35–2.18), compared to referent. By contrast, there were no associations for the following groups: those with an elevated biomarker ratio without MetS (elevated BUN-CR: HR = 1.05, 0.79–1.39; elevated GGT-ALP: HR = 1.24, 0.92–1.67), MetS without elevated biomarker ratio (normoalbuminuria: HR = 1.05, 0.89–1.24; BUN-CR: HR = 1.10, 0.93–1.31; GGT-ALP: HR = 1.12, 0.96–1.32; AST-ALT: HR = 1.17, 1.00–1.38), and MetS with elevated biomarker ratio (elevated BUN-CR: HR = 1.26, 0.96–1.64; elevated GGT-ALP: HR = 1.26, 0.95–1.67), compared to referent. In a subsample without self-reported comorbidities (CVD, diabetes, or hypertension), the relationships in the fully adjusted (for covariates) model were maintained (Table 2); in addition, there was a statistically significant association between elevated GGT-ALP with MetS (HR = 1.39, 1.02–1.90) and all-cause mortality, compared to referent.

**Table 2:** Association between all-cause mortality and kidney or liver biomarker ratio with or without MetS (referent)

Biomarkers	Groups		Unadjusted^a^		Adjusted^b^		Adjusted^c^	
	MetS status	High biomarker ratio	Hazard ratios^a^	95% confidence interval	Hazard ratios^b^	95% confidence interval	Hazard ratios^c^	95% confidence interval
UACR	No	No	1.00 (REF)	–	1.00 (REF)	–	1.00 (REF)	–
	No	Yes	4.12	3.16 to 5.37	2.12	1.65 to 2.72	2.07	1.53 to 2.79
	Yes	No	1.93	1.64 to 2.26	1.05	0.89 to 1.24	1.04	0.86 to 1.24
	Yes	Yes	6.11	4.78 to 7.80	2.57	1.99 to 3.33	2.40	1.76 to 3.26
BUN-CR	No	No	1.00 (REF)	–	1.00 (REF)	–	1.00 (REF)	–
	No	Yes	1.63	1.23 to 2.16	1.05	0.79 to 1.39	0.99	0.71 to 1.38
	Yes	No	2.02	1.71 to 2.39	1.10	0.93 to 1.31	1.09	0.90 to 1.31
	Yes	Yes	3.38	2.66 to 4.28	1.26	0.96 to 1.64	1.12	0.82 to 1.54
GGT-ALP	No	No	1.00 (REF)	–	1.00 (REF)	–	1.00 (REF)	–
	No	Yes	1.45	1.06 to 1.99	1.24	0.92 to 1.67	1.21	0.88 to 1.65
	Yes	No	2.17	1.87 to 2.53	1.12	0.96 to 1.32	1.07	0.90 to 1.28
	Yes	Yes	2.07	1.54 to 2.77	1.26	0.95 to 1.67	1.39	1.02 to 1.90
AST-ALT	No	No	1.00 (REF)	–	1.00 (REF)	–	1.00 (REF)	–
	No	Yes	2.25	1.78 to 2.85	1.71	1.35 to 2.18	1.69	1.30 to 2.21
	Yes	No	2.19	1.88 to 2.55	1.17	1.00 to 1.38	1.15	0.97 to 1.37
	Yes	Yes	6.71	4.83 to 9.34	2.22	1.61 to 3.07	2.09	1.46 to 3.00

Data derived from NHANES 1999–2015; referent group has neither elevated biomarker ratio nor MetS. Covariates include age, sex, ethnicity, education, income ratio, alcohol use, smoking, hospital visits, insurance coverage, self-rated health, and physical activity

^a^Unadjusted model of the analytic subsample (n = 10,604); n = 1215 deaths

^b^Fully adjusted model of the analytic subsample (n = 10,604); n = 1215 deaths

^c^Fully adjusted model of a subsample without self-reported CVD, diabetes, or hypertension (n = 9909); n = 970 deaths

In fully adjusted models using prevalent MetS as the referent group (Table 3) there was a positive association between elevated biomarker ratio without MetS (elevated UACR: HR = 2.03, 1.56–2.63, and elevated AST-ALT: HR = 1.46, 1.13–1.89), and elevated biomarker ratio with MetS (elevated UACR: HR = 2.46, 1.96–3.11, and elevated AST-ALT: HR = 1.89, 1.40–2.57), and all-cause mortality. No associations were observed for the following groups: neither elevated biomarker ratio nor MetS, and; elevated biomarker ratio with/without MetS (BUN-CR and GGT-ALP).

**Table 3:** Association between all-cause mortality and kidney or liver biomarker ratio without or with MetS (referent)

Biomarkers	Groups		Unadjusted^a^		Adjusted^b^		Adjusted^c^	
	MetS status	High biomarker ratio	Hazard ratios^a^	95% confidence interval	Hazard ratios^b^	95% confidence interval	Hazard ratios^c^	95% confidence interval
UACR	Yes	No	1.00 (REF)	–	1.00 (REF)	–	1.00 (REF)	–
	No	No	0.52	0.44 to 0.61	0.96	0.81 to 1.13	0.97	0.80 to 1.16
	No	Yes	2.13	1.65 to 2.77	2.03	1.56 to 2.63	2.00	1.49 to 2.68
	Yes	Yes	3.17	2.54 to 3.95	2.46	1.94 to 3.11	2.32	1.76 to 3.05
BUN-CR	Yes	No	1.00 (REF)	–	1.00 (REF)	–	1.00 (REF)	–
	No	No	0.50	0.42 to 0.59	0.91	0.77 to 1.08	0.92	0.76 to 1.11
	No	Yes	0.81	0.61 to 1.06	0.95	0.71 to 1.27	0.91	0.66 to 1.26
	Yes	Yes	1.67	1.29 to 2.16	1.14	0.88 to 1.48	1.04	0.75 to 1.43
GGT-ALP	Yes	No	1.00 (REF)	–	1.00 (REF)	–	1.00 (REF)	–
	No	No	0.46	0.40 to 0.54	0.89	0.76 to 1.04	0.93	0.78 to 1.12
	No	Yes	0.67	0.49 to 0.92	1.10	0.80 to 1.52	1.13	0.80 to 1.59
	Yes	Yes	0.95	0.73 to 1.25	1.12	0.85 to 1.48	1.30	0.95 to 1.78
AST-ALT	Yes	No	1.00 (REF)	–	1.00 (REF)	–	1.00 (REF)	–
	No	No	0.46	0.39 to 0.53	0.85	0.73 to 1.00	0.87	0.73 to 1.04
	No	Yes	1.03	0.79 to 1.34	1.46	1.13 to 1.89	1.47	1.11 to 1.96
	Yes	Yes	3.07	2.20 to 4.27	1.89	1.40 to 2.57	1.82	1.28 to 2.57

Data derived from NHANES 1999–2015; referent group has prevalent MetS. Covariates include age, sex, ethnicity, education, income ratio, alcohol use, smoking, hospital visits, insurance coverage, self-rated health, and physical activity

^a^Unadjusted model of the analytic subsample (n = 10,604); n = 1215 deaths

^b^Fully adjusted model of the analytic subsample (n = 10,604); n = 1215 deaths

^c^Fully adjusted model of a subsample without self-reported CVD, diabetes, or hypertension (n = 9909); n = 970 deaths

### CHD mortality

Detailed analysis of the relationship between CHD mortality and co-occurrence of MetS with kidney, or liver, biomarker ratios are presented in the additional tables. In fully adjusted models (Fig. 2), CHD mortality was positively associated with the following groups: elevated biomarker ratio with MetS (elevated UACR: HR = 1.67, 1.05–2.67, and elevated AST-ALT: HR = 2.80, 1.62–4.86), and elevated BUN-CR without MetS (HR = 2.12, 1.12–4.04), compared to the referent group with neither elevated biomarker ratio nor MetS. No associations were observed for groups with elevated biomarker ratio without MetS (elevated UACR: HR = 1.22, 0.66–2.24; GGT-ALP: HR = 1.60, 0.80–3.21; AST-ALT: HR = 1.40, 0.76–2.58), MetS without elevated biomarker ratio (normoalbuminuria: HR = 0.83, 0.54–1.26; BUN-CR: HR = 1.17, 0.77–1.77; GGT-ALP: HR = 1.11, 0.77–1.61; AST-ALT: HR = 0.88, 0.62–1.24), or MetS with elevated biomarker ratio (BUN-CR: HR = 0.81, 0.40–1.64; GGT-ALP: HR = 0.81, 0.45–1.48), compared to referent. In a subsample without self-reported comorbidities (CVD, diabetes, or hypertension), only MetS with elevated AST-ALT (HR = 3.11, 1.49–6.49) was positively associated with CHD mortality (Table 4).

**Table 4:** Association between CHD mortality and kidney or liver biomarker ratio with or without MetS (referent)

Biomarkers	Groups		Unadjusted^a^		Adjusted^b^		Adjusted^c^	
	MetS status	High biomarker ratio	Hazard ratios^a^	95% confidence interval	Hazard ratios^b^	95% confidence interval	Hazard ratios^c^	95% confidence interval
UACR	No	No	1.00 (REF)	–	1.00 (REF)	–	1.00 (REF)	–
	No	Yes	1.92	1.03 to 3.61	1.22	0.66 to 2.24	0.67	0.33 to 1.39
	Yes	No	0.97	0.64 to 1.47	0.83	0.54 to 1.26	0.77	0.46 to 1.31
	Yes	Yes	1.76	1.02 to 3.02	1.67	1.05 to 2.67	1.71	0.95 to 3.09
BUN-CR	No	No	1.00 (REF)	–	1.00 (REF)	–	1.00 (REF)	–
	No	Yes	1.43	0.74 to 2.77	2.12	1.12 to 4.04	1.90	0.84 to 4.29
	Yes	No	1.20	0.78 to 1.83	1.17	0.77 to 1.77	1.26	0.76 to 2.09
	Yes	Yes	0.79	0.44 to 1.44	0.81	0.40 to 1.64	0.59	0.22 to 1.55
GGT-ALP	No	No	1.00 (REF)	–	1.00 (REF)	–	1.00 (REF)	–
	No	Yes	1.87	0.87 to 4.03	1.60	0.80 to 3.21	1.42	0.58 to 3.45
	Yes	No	1.15	0.79 to 1.68	1.11	0.77 to 1.61	1.19	0.74 to 1.93
	Yes	Yes	1.11	0.59 to 2.09	0.81	0.45 to 1.48	0.70	0.33 to 1.47
AST-ALT	No	No	1.00 (REF)	–	1.00 (REF)	–	1.00 (REF)	–
	No	Yes	1.25	0.69 to 2.26	1.40	0.76 to 2.58	1.52	0.74 to 3.11
	Yes	No	0.94	0.65 to 1.35	0.88	0.62 to 1.24	0.92	0.59 to 1.45
	Yes	Yes	2.86	1.58 to 5.19	2.80	1.62 to 4.86	3.11	1.49 to 6.49

Data derived from NHANES 1999–2015; referent group has neither elevated biomarker ratio nor MetS. Covariates include age, sex, ethnicity, education, income ratio, alcohol use, smoking, hospital visits, insurance coverage, self-rated health, and physical activity

^a^Unadjusted model of the analytic subsample (n = 1212); n = 201 deaths

^b^Fully adjusted model of the analytic subsample (n = 1212); n = 201 deaths

^c^Fully adjusted model of a subsample without self-reported CVD, diabetes, or hypertension (n = 968); n = 134 deaths

In a final set of models (unadjusted and adjusted for covariates), we explored the contribution of MetS to CHD mortality using prevalent MetS as the referent group (Table 5) and found a positive association between CHD mortality and elevated biomarker ratio with MetS (elevated UACR: HR = 2.03, 1.28–3.22, and elevated AST-ALT: HR = 3.20, 1.86–5.51). No other subgroups were related to CHD mortality.

**Table 5:** Association between CHD mortality and kidney or liver biomarker ratio without or with MetS (referent)

Biomarkers	Groups		Unadjusted^a^		Adjusted^b^		Adjusted^c^	
	MetS status	High biomarker ratio	Hazard ratios^a^	95% confidence interval	Hazard ratios^b^	95% confidence interval	Hazard ratios^c^	95% confidence interval
UACR	Yes	No	1.00 (REF)	–	1.00 (REF)	–	1.00 (REF)	–
	No	No	1.03	0.68 to 1.56	1.21	0.79 to 1.85	1.30	0.77 to 2.19
	No	Yes	1.98	1.14 to 3.44	1.48	0.81 to 2.69	0.87	0.41 to 1.87
	Yes	Yes	1.81	1.11 to 2.96	2.03	1.28 to 3.22	2.22	1.24 to 3.97
BUN-CR	Yes	No	1.00 (REF)	–	1.00 (REF)	–	1.00 (REF)	–
	No	No	0.84	0.55 to 1.28	0.86	0.57 to 1.29	0.79	0.48 to 1.31
	No	Yes	1.20	0.63 to 2.27	1.82	1.00 to 3.29	1.50	0.67 to 3.35
	Yes	Yes	0.66	0.32 to 1.36	0.69	0.32 to 1.49	0.47	0.17 to 1.28
GGT-ALP	Yes	No	1.00 (REF)	–	1.00 (REF)	–	1.00 (REF)	–
	No	No	0.87	0.60 to 1.26	0.90	0.62 to 1.31	0.84	0.52 to 1.35
	No	Yes	1.62	0.79 to 3.34	1.45	0.78 to 2.68	1.19	0.51 to 2.78
	Yes	Yes	0.96	0.49 to 1.87	0.73	0.42 to 1.29	0.58	0.31 to 1.11
AST-ALT	Yes	No	1.00 (REF)	–	1.00 (REF)	–	1.00 (REF)	–
	No	No	1.07	0.74 to 1.54	1.40	0.76 to 2.58	1.09	0.69 to 1.71
	No	Yes	1.33	0.73 to 2.45	0.88	0.62 to 1.24	1.65	0.75 to 3.62
	Yes	Yes	3.06	1.73 to 5.42	3.20	1.86 to 5.51	3.38	1.64 to 6.98

Data derived from NHANES 1999–2015; referent group has prevalent MetS. Covariates include age, sex, ethnicity, education, income ratio, alcohol use, smoking, hospital visits, insurance coverage, self-rated health, and physical activity

^a^Unadjusted model of the analytic subsample (n = 1212); n = 201 deaths

^b^Fully adjusted model of the analytic subsample (n = 1212); n = 201 deaths

^c^Fully adjusted model of a subsample without self-reported CVD, diabetes, or hypertension (n = 968); n = 134 deaths

### Missing analysis

The extent of missing covariate data was evaluated in a sample (n = 49,512) without individuals < 20 years, and the analytic sample (n = 13,731). Overall, no differences in socio-demographics, behavioral factors, or healthcare utilization and insurance coverage were found between the full and analytic sample.

## Discussion

The present study was an analysis of adults without self-identified kidney or liver conditions in the United States from 1999 to 2016. We aimed to identify the relationship between well-known surrogates of kidney and liver dysfunction with (and without) MetS, and all-cause, and CHD mortality. Among the one-third of U.S. adults who have MetS, most biomarker ratios tended to be higher than that of individuals without MetS. Positive associations with all-cause mortality were observed for elevated UACR or albuminuria, and elevated AST-ALT, both with and without MetS. Positive associations with CHD mortality were observed for elevated UACR, and elevated AST-ALT, with MetS. As well, positive associations with CHD mortality were observed for elevated BUN-CR without MetS, and; positive associations with all-cause mortality for elevated GGT-ALP with MetS in participants without CVD, diabetes, or hypertension.

All-cause/CHD mortality was not associated with prevalent MetS (without elevated biomarker ratios), nor groups with neither elevated biomarker ratio nor MetS. As an intermediary step, a sensitivity analysis was performed (see Additional file 1: Table S1) in a fully adjusted logistic regression of the odds between the combination of MetS with elevation in biomarker ratios on CVD, which did not change the relationship.

### Clinical application

Similar to the findings from this study, the presence of elevated kidney or liver biomarkers has previously been found to be associated with a higher risk of all-cause [18, 20, 22, 34], or cardiovascular mortality [17, 19, 24, 25]. Furthermore, a UACR or BUN-CR test enables the prediction of renal dysfunction or nephropathy [27, 42], including the evaluation of CVD events [8, 9, 18–21, 38]. In terms of the surrogates for liver dysfunction, there is no single biomarker that can definitively diagnose or distinguish diseases of the liver [5, 6, 22, 23, 43]. Currently, GGT on its own has limited specificity [6, 22], and is known to be sensitive to variations in alcohol intake [42]; however, GGT-ALP could help to ascertain whether the source of a disease could be due to liver or bone pathology [22].

Routine measurements of AST and ALT support the detection of liver dysfunction [5, 6, 44]. ALT activity, however, is specific to the liver, while AST is systemic and can be found in the brain, heart, liver, lungs, kidneys, skeletal muscles, and pancreas [42]. Although AST is a less specific indicator of hepatic injury than ALT, as a ratio—AST-ALT—they tend to elevate simultaneously in cases of hepatitis, cirrhosis, hepatobiliary disorders, or necroinflammation [5, 42, 44]. In this analysis, we observed that elevated AST-ALT with and without MetS was positively associated with all-cause mortality, and; positively associated with CHD mortality in the presence of MetS, while elevated GGT-ALP with MetS was positively associated with all-cause mortality.

Evidence in support of the use of elevated AST-ALT ratio in mortality, MetS, or its comorbid risk factors is mixed, and an area of ongoing research [22, 24, 29, 45–47]. In MetS positive cases, the AST-ALT ratio tends to be lower, which is consistent with previous studies [15, 29]. Insulin resistance, fat infiltration of the liver, or visceral adiposity [15], and medical prescriptions [24] may explain the relationship. Studies [6, 15, 24, 25] have also reported a long-term risk for metabolic disorders with normal or abnormal ALT. Taken together, however, elevated AST-ALT alongside elevated GGT or ALP has been shown to improve the identification of severe cardiometabolic disease [5, 48, 49]. Further studies are needed to investigate the potential of these biomarkers and their appropriate reference values to predict population health risks more accurately [6, 16, 22, 24, 25, 34], and may be particularly valuable for risk stratification of obesity phenotypes [47].

### Strengths and limitations

The main advantages of this study were: its multicycle cross-sectional design and mortality linkage, which allowed for the determination of prevalence of elevated biomarker levels and 6.9 year average survival estimates with adjustment for a host of potential confounders. The main limitations of this study were the single baseline measure of analytes and that directionality and causality could not be determined for the inter-relationship between biomarkers and MetS. Despite a large representative sample, our analyses were limited to thresholds of biomarker ratios, rather than a full examination of dose–response patterns.

## Conclusion

This study is among a few studies to use a national representative sample of the U.S. adult population to compare the effects of biomarkers on MetS, and their related effects on all-cause and CHD mortality. MetS with albuminuria or with elevated AST-ALT was positively related to CHD mortality, while albuminuria, and elevated AST-ALT, with and without MetS, were positively related to all-cause death. Further work is therefore necessary to understand the utility of kidney and liver biomarker ratios in the prediction of all-cause and cause-specific mortality. Future longitudinal studies should make available the in-depth tracking and analyses of the long-term impact of these components in subpopulations of at-risk adults in order to improve risk stratification for chronic disease management.

## Supplementary Information

**Additional file 1: Table S1**. Association between cardiovascular disease and kidney or liver biomarker ratio with or without MetS. Data derived from NHANES 1999–2015; referent group has neither elevated biomarker ratio nor MetS. Unadjusted and Fully adjusted models of the analytic subsample. Covariates include age, sex, ethnicity, education, income ratio, alcohol use, smoking, hospital visits, insurance coverage, self-rated health, and physical activity.

## Data Availability

The datasets generated and/or analysed during the current study are available from the U.S. Department of Health and Human Services, Centers for Disease Control and Prevention [https://wwwn.cdc.gov/nchs/nhanes]. Public-use Linked Mortality Files (LMF) is available for the 1999–2014 NHANES [https://www.cdc.gov/nchs/data-linkage/mortality-public.htm].

## Abbreviations

CHD: Coronary heart disease
NHANES: National health and nutrition examination survey
CVD: Cardiovascular disease
ALT: Alanine aminotransferase
AST: Aspartate aminotransferase
ALP: Alkaline phosphatase
GGT: Gamma-glutamyl transferase
NAFLD: Non-alcoholic fatty liver disease
ALD: Alcoholic liver disease
LMF: Linked mortality files
CV: Coefficient of variation
BUN: Blood urea nitrogen
CR: Serum creatinine
HDL: High density lipoprotein
UA: Urinary albumin
UCR: Urinary creatinine
NDI: National death index
NCHS: National center for health statistics
PA: Physical activity
MET: Metabolic equivalent task
ICD: International classification of diseases
