# Supplementary material for: Associations between elevated kidney and liver biomarker ratios, metabolic syndrome and all-cause and coronary heart disease (CHD) mortality: analysis of the U.S. National Health and Nutrition Examination Survey (NHANES)
Source: BMC Cardiovasc Disord. 2021 Jul 26;21:352. doi: 10.1186/s12872-021-02160-w (PMC8311936; doi:10.1186/s12872-021-02160-w)
Supplement: Supplementary file 1 — Additional file 1: Table S1. Association between cardiovascular disease and kidney or liver biomarker ratio with or without MetS. Data derived from NHANES 1999–2015; referent group has neither elevated biomarker ratio nor MetS. Unadjusted and Fully adjusted models of the analytic subsample. Covariates include age, sex, ethnicity, education, income ratio, alcohol use, smoking, hospital visits, insurance coverage, self-rated health, and physical activity. [file 12872_2021_2160_MOESM1_ESM.docx]

| **Table S1**. Association between cardiovascular disease and kidney or liver biomarker ratio with or without MetS. | | | | | | |
| --- | --- | --- | --- | --- | --- | --- |
| Biomarkers | Groups | | Unadjusted*^1^* | | Adjusted*^2^* | |
|  | MetS Status | High Status | Hazard Ratios*^1^* | 95% Confidence Interval | Hazard Ratios*^2^* | 95% Confidence Interval |
| UACR | No | No | 1.00 (REF) | - | 1.00 (REF) | - |
|  | No | Yes | 2.85 | 2.15 to 3.80 | 1.53 | 1.08 to 2.16 |
|  | Yes | No | 2.25 | 1.88 to 2.70 | 1.32 | 1.09 to 1.59 |
|  | Yes | Yes | 4.62 | 3.46 to 6.18 | 1.93 | 1.39 to 2.69 |
|  |  |  |  |  |  |  |
| BUN-CR | No | No | 1.00 (REF) | - | 1.00 (REF) | - |
|  | No | Yes | 1.66 | 1.17 to 2.35 | 1.13 | 0.77 to 1.66 |
|  | Yes | No | 2.31 | 1.89 to 2.83 | 1.33 | 1.08 to 1.63 |
|  | Yes | Yes | 3.44 | 2.55 to 4.66 | 1.60 | 1.12 to 2.27 |
|  |  |  |  |  |  |  |
| GGT-ALP | No | No | 1.00 (REF) | - | 1.00 (REF) | - |
|  | No | Yes | 1.05 | 0.71 to 1.55 | 0.83 | 0.56 to 1.22 |
|  | Yes | No | 2.23 | 1.86 to 2.67 | 1.26 | 1.05 to 1.53 |
|  | Yes | Yes | 2.81 | 1.99 to 3.96 | 1.61 | 1.10 to 2.37 |
|  |  |  |  |  |  |  |
| AST-ALT | No | No | 1.00 (REF) | - | 1.00 (REF) | - |
|  | No | Yes | 1.11 | 0.82 to 1.50 | 0.84 | 0.60 to 1.16 |
|  | Yes | No | 2.28 | 1.90 to 2.73 | 1.31 | 1.08 to 1.59 |
|  | Yes | Yes | 3.49 | 2.07 to 5.86 | 1.20 | 0.70 to 2.06 |
|  |  |  |  |  |  |  |
| Data derived from NHANES 1999-2016; referent group has neither elevated biomarker ratio nor MetS. | | | | | | |
| *^1^* Unadjusted model of the analytic sample (n=13731); n=877 cases. | | | | | | |
| *^2^* Fully adjusted model of the analytic sample (n=13731); n=877 cases. | | | | | | |
| Covariates include age, sex, ethnicity, education, income ratio, alcohol use, smoking, hospital visits, insurance coverage, self-rated health, and physical activity. | | | | | | |
